# Renal function during hospitalization and outcome in Chinese patients with acute decompensated heart failure: A retrospective study and literature review

**DOI:** 10.1002/clc.23934

**Published:** 2022-11-07

**Authors:** Hao‐Wei Lee, Chin‐Chou Huang, Chih‐Yu Yang, Hsin‐Bang Leu, Po‐Hsun Huang, Tao‐Cheng Wu, Shing‐Jong Lin, Jaw‐Wen Chen

**Affiliations:** ^1^ Division of Cardiology, Department of Medicine Taipei Veterans General Hospital Taipei Taiwan; ^2^ School of Medicine National Yang Ming Chiao Tung University Taipei Taiwan; ^3^ Institute of Pharmacology National Yang Ming Chiao Tung University Taipei Taiwan; ^4^ Cardiovascular Research Center National Yang Ming Chiao Tung University Taipei Taiwan; ^5^ Division of Nephrology, Department of Medicine Taipei Veterans General Hospital Taipei Taiwan; ^6^ Institute of Clinical Medicine National Yang Ming Chiao Tung University Taipei Taiwan; ^7^ Healthcare and Services Center Taipei Veterans General Hospital Taipei Taiwan; ^8^ Department of Critical Care Medicine Taipei Veterans General Hospital Taipei Taiwan; ^9^ Taipei Heart Institute Taipei Medical University Taipei Taiwan

**Keywords:** acute decompensated heart failure, cardiovascular event, hospitalization, renal function, worsening renal function

## Abstract

**Background:**

The heart and kidneys had demonstrated a bidirectional interaction that dysfunction of the heart or kidneys can induce dysfunction in the other organ.

**Hypothesis:**

Renal function and its decline during hospitalization may have impact on cardiovascular outcomes in patients with acute decompensated heart failure (ADHF).

**Methods:**

A total of 119 consecutive Chinese patients admitted for ADHF were prospectively enrolled. The course of renal function was presented with estimated glomerular filtration rate (eGFR), calculated by the four‐variable equation proposed by the Modification of Diet in Renal Disease (MDRD) Study. Worsening renal function (WRF) was defined as eGFR decline between admission (eGFR_admission_) and predischarge (eGFR_predischarge_). Clinical outcomes were defined as 4P‐major adverse cardiovascular events (4P‐MACE), including the composition of cardiovascular death, nonfatal myocardial infarction, nonfatal stroke, and nonfatal HF hospitalization.

**Results:**

During an average 2.6 ± 3.2 years follow‐up, 66 patients (55%) experienced 4P‐MACE. Patients with impaired eGFR_predischarge_ (<60 ml/min/1.73 m^2^) had more 4P‐MACE than those with preserved eGFR_predischarge_ (64.7% vs. 43.1%, *p* = .019). The Kaplan–Meier survival curves showed significantly higher incidence of 4P‐MACE in patients with impaired eGFR_predischarge_ than those with preserved eGFR_predischarge_ (*p* = .002). Cox regression analysis revealed that impaired eGFR_predischarge_ was significantly correlated with the development of 4P‐MACE (hazard ratio, 2.003; 95% confidence interval, 1.072–3.744; *p* = .029). In contrast, outcomes would be similar with regard to eGFR on admission and eGFR decline during hospitalization.

**Conclusions:**

Impaired renal function before discharge, but not impaired renal function on admission or WRF, is a significant risk factor for poor outcomes in patients with ADHF.

## 1 INTRODUCTION

It is well known that the heart and kidney have a bidirectional correlation, in which one organ dysfunction results in maladaptive change in the other.^1^ The prevalence of renal insufficiency ranges from 20% to 57% in patients with chronic stable heart failure (HF) and acute decompensated heart failure (ADHF).^2^ Compared to patients without chronic kidney disease (CKD), patients with both HF and CKD may have up to a 50% greater risk of mortality, especially patients of HF with reduced ejection fraction (HFrEF).^3^ In addition to mortality, patients with CKD hospitalized with HF also have a greater risk of CKD progression.^4^

The impact of worsening renal function (WRF) on the clinical outcomes in patients with ADHF remains controversial. Some studies have shown that WRF in patients admitted due to ADHF was associated with higher in‐hospital mortality, longer hospital stay, readmission, higher mortality after discharge, and higher costs.^5^, ^6^, ^7^, ^8^, ^9^, ^10^, ^11^, ^12^, ^13^ However, some studies have shown that patients with WRF have similar mortality and rehospitalization rates to those without WRF.^14^, ^15^, ^16^, ^17^, ^18^ Furthermore, there is scarce information regarding the impact of WRF in Chinese patients. Therefore, we aimed to investigate the impact of renal function and its changes during hospitalization on clinical outcomes in Chinese patients admitted for ADHF.

## 2 METHODS

### 2.1 Participants

Consecutive Chinese patients admitted for ADHF were enrolled at the Taipei Veterans General Hospital between June 1, 2008 and April 31, 2010. ADHF was defined according to current guidelines.^19^, ^20^ Patients with severe comorbidity, end‐stage renal disease (ESRD), moderate or severe pulmonary disease, malignancy, uncontrolled thyroid disease, drug or alcohol abuse, infections, or an inflammatory illness such as sepsis, arthritis, or connective tissue disease were excluded. Patients with acute coronary syndrome, significant aortic valve disease, myocarditis, infiltrative or hypertrophic cardiomyopathy, uncontrolled tachyarrhythmias, in need of a mechanical assist device, or having significant congenital heart disease were also excluded. The study protocol was approved by the Ethics Committee of Taipei Veterans General Hospital. All patients agreed to participate and signed the informed consent document for the study. This study was conducted in accordance with the principles of the Declaration of Helsinki.

### 2.2 Study design

The study included a comprehensive examination of each participant's patient history and physical examination. Patient comorbidities were recorded, including hypertension (HTN), diabetes mellitus (DM), atrial fibrillation (AF), and hyperlipidemia. Patients were defined as having ischemic heart disease (IHD) if their coronary angiography showed a ≥70% luminal diameter and narrowing in at least one major epicardial coronary artery, or if there was documented myocardial infarction or HF secondary to post‐infarction ventricular aneurysm. Echocardiography was performed, and left ventricular ejection fraction (LVEF) was measured. Patients with LVEF <40% were defined as having HFrEF.

Predischarge medication prescriptions for HF were recorded, and these included diuretics, angiotensin‐converting enzyme inhibitors (ACEI)/angiotensin receptor blockers (ARB), beta‐blockers, digitalis, and vasodilators.

### 2.3 Laboratory measurements

Fasting whole‐blood samples of the patients were obtained by venipuncture. The blood samples were centrifuged, and the serum was used for analysis. Cardiac troponin‐I levels were determined using an Abbott Axsym system (Abbott Laboratories). The N‐terminal pro‐brain natriuretic peptide (NT‐pro‐BNP) was measured using the Roche Elecsys NT‐proBNP (Roche Diagnostics GmbH). High‐sensitivity C‐reactive protein (hs‐CRP) levels were determined with a validated, high‐sensitivity assay using an autoanalyzer (IMMAGE Immunochemistry Systems, Beckman Coulter, Inc.).

A series of studies on serum creatinine levels during hospitalization were performed. Estimated glomerular filtration rate (eGFR) was calculated using the four‐variable equation proposed by the Modification of Diet in Renal Disease (MDRD) Study.^21^ Patients were further divided into different groups according to eGFR at admission (eGFR_admission_), eGFR at predischarge (eGFR_predischarge_), and eGFR change between admission and predischarge. According to eGFR_admission_ levels, patients were divided into the preserved eGFR_admission_ group (≥60 ml/min/1.73 m^2^) and impaired eGFR_admission_ group (<60 ml/min/1.73 m^2^). According to eGFR_predischarge_ levels, patients were divided into the preserved eGFR_predischarge_ group (≥60 ml/min/1.73 m^2^) and impaired eGFR_predischarge_ group (<60 ml/min/1.73 m^2^). WRF was defined as an eGFR decline between admission and predischarge.

### 2.4 Clinical outcomes

Clinical outcomes during the follow‐up period were defined as 4P‐major adverse cardiovascular events (4P‐MACE), including the composition of cardiovascular (CV) death, nonfatal myocardial infarction (MI), nonfatal stroke, and nonfatal HF hospitalization.

### 2.5 Statistical analysis

Statistical analysis was performed using the Statistical Package for Social Sciences software (version 21.0, SPSS Inc.). All data are expressed as the mean ± SD or frequency (percentage). Parametric continuous data between different patient groups were compared using the unpaired Student's *t*‐test, and nonparametric data were compared using the Mann–Whitney test. Categorical variables were analyzed using the *χ* ^2^‐squared test or Fisher's exact test. Survival analysis was assessed using the Kaplan–Meier curve, with significance based on the log‐rank test. To assess the independent effects of renal function (impaired eGFR_admission_, impaired eGFR_predischarge_, and eGFR decline) and 4P‐MACE, Cox proportional hazard regression analysis was performed. The adjusted hazard ratios (HRs) with 95% confidence intervals (CIs) were estimated after adjusting for potential confounding factors. The HRs of renal function for 4P‐MACE were adjusted for age, sex, HTN, DM, ischemic heart disease (IHD), and predischarge medications for HF. Statistical significance was defined as a two‐sided *p* < .05.

## 3 RESULTS

### 3.1 Baseline characteristics

A total of 119 patients with ADHF were eligible for enrollment. The mean age of the participants was 73.5 ± 12.9 years, and approximately 76.5% were men. The average body mass index was 23.9 ± 4.5 kg/m^2^. The comorbidities included HTN in 92 patients (77.3%), DM in 48 patients (40.3%), AF in 57 patients (47.9%), and hyperlipidemia in 29 patients (24.4%). Among these patients, 60 (50.4%) had ischemic heart disease. There were 58 patients (48.7%) with HFrEF, and the mean LVEF was 40.3 ± 15.5%. Predischarge medications included diuretics (79.8%), ACEI/ARB (71.4%), beta‐blockers (47.1%), digitalis (22.7%), and vasodilators (52.9%). The renal function of the participants upon admission was a serum creatinine level of 1.7 ± 0.9 mg/dl and an eGFR_admission_ of 50.7 ± 25.9 ml/min/1.73 m^2^. The renal function of the participants before discharge was a serum creatinine level of 1.5 ± 0.7 mg/dl and an eGFR_predischarge_ of 59.2 ± 31.3 ml/min/1.73 m^2^. The average change in eGFR was 0.2 ± 0.5 ml/min/1.73 m^2^ (Table 1).

**Table 1:** Baseline characteristics of the patients

	All (*n* = 119)
Age, years	73.5 ± 12.9
Male, *n* (%)	91 (76.5%)
BMI, kg/m^2^	23.9 ± 4.5
Smoking, *n* (%)	27 (22.7%)
HTN, *n* (%)	92 (77.3%)
DM, *n* (%)	48 (40.3%)
AF, *n* (%)	57 (47.9%)
Hyperlipidemia, *n* (%)	29 (24.4%)
IHD, *n* (%)	60 (50.4%)
HFrEF, *n* (%)	58 (48.7%)
LVEF, %	40.3 ± 15.5
Laboratory data	
HDL‐C, mg/dl	43.2 ± 19.0
LDL‐C, mg/dl	88.4 ± 30.3
hs‐CRP, mg/dl	1.2 ± 1.4
Troponin‐I, ng/ml	0.1 ± 0.2
NT‐pro‐BNP, pg/ml	4006.2 ± 4036.4
Creatinine_admission_, mg/dl	1.7 ± 0.9
Creatinine_predischarge_, mg/dl	1.5 ± 0.7
eGFR_admission_, ml/min/1.73 m^2^	50.7 ± 25.9
eGFR_predischarge_, ml/min/1.73 m^2^	59.2 ± 31.3
eGFR change, ml/min/1.73 m^2^	0.2 ± 0.5
Predischarge medication	
Diuretics, *n* (%)	95 (79.8%)
ACEI/ARB, *n* (%)	85 (71.4%)
Beta‐blockers, *n* (%)	56 (47.1%)
Digitalis, *n* (%)	27 (22.7%)
Vasodilators, *n* (%)	63 (52.9%)
Follow‐up duration (years)	2.6 ± 3.2

Abbreviations: ACEI, angiotensin‐converting enzyme inhibitor; AF, atrial fibrillation; ARB, angiotensin receptor blocker; BMI, body mass index; DM, diabetes mellitus; eGFR, estimated glomerular filtration rate; HDL‐C, high‐density lipoprotein‐cholesterol; HFrEF, heart failure with reduced ejection fraction; HTN, hypertension; hs‐CRP, high sensitivity C‐reactive protein; IHD, ischemic heart disease; LDL‐C, low‐density lipoprotein‐cholesterol; LVEF, left ventricular ejection fraction; NT‐pro‐BNP, N‐terminal pro‐brain natriuretic peptide.

Based on renal function upon admission, there were 35 patients with preserved eGFR_admission_ (≥60 ml/min/1.73 m^2^) and 84 patients with impaired eGFR_admission_ (<60 ml/min/1.73 m^2^). When comparing to those with preserved eGFR_admission_, patients with impaired eGFR_admission_ had more HTN (83.3% vs. 62.9%, *p* = .015), DM (47.6% vs. 22.9%, *p* = .012), and ischemic heart disease (57.1% vs. 34.3%, *p* = .023), and used more vasodilators than patients with preserved eGFR_admission_ (59.5% vs. 37.1%, *p* = .026). The mean eGFR_admission_ was 37.9 ± 13.3 ml/min/1.73 m^2^ in patients with impaired eGFR_admission_ and 81.3 ± 23.1 ml/min/1.73 m^2^ in patients with preserved eGFR_admission_ (*p* < .001). The mean eGFR_predischarge_ was 46.6 ± 20.4 ml/min/1.73 m^2^ in patients with impaired eGFR_admission_ and 89.5 ± 32.5 ml/min/1.73 m^2^ in patients with preserved eGFR_admission_ (*p* < .001). The changes in eGFR between admission and discharge were similar in the two groups (Supporting Information: Table 1).

According to the renal function before discharge, 51 patients had preserved eGFR_predischarge_ (≥60 ml/min/1.73 m^2^) and 68 patients with impaired eGFR_predischarge_ (<60 ml/min/1.73 m^2^). Compared to patients with preserved eGFR_predischarge_, patients with impaired eGFR_predischarge_ had more DM (51.5% vs. 25.5%, *p* = .004) and ischemic heart disease (58.8% vs. 39.2%, *p* = .034), and used more diuretics (86.8% vs. 70.6%, *p* = .030) and less digitalis (11.8% vs. 37.3%, *p* = .001) than patients with preserved eGFR_predischarge_. The mean eGFR_admission_ was 37.2 ± 14.5 ml/min/1.73 m^2^ in patients with impaired eGFR_predischarge_ and 68.6 ± 27.0 ml/min/1.73 m^2^ in patients with preserved eGFR_predischarge_ (*p* < .001). The mean eGFR_predischarge_ was 39.0 ± 12.5 ml/min/1.73 m^2^ in patients with impaired eGFR_predischarge_ and 86.2 ± 28.5 ml/min/1.73 m^2^ in patients with preserved eGFR_predischarge_ (*p* < .001). Patients with preserved eGFR_predischarge_ showed significantly more improvement in eGFR than those with impaired eGFR_predischarge_ (0.4 ± 0.6 ml/min/1.73 m^2^ vs. 0.1 ± 0.4 ml/min/1.73 m^2^, *p* = .012; Table 2).

**Table 2:** Baseline characteristics according to predischarge renal function

	Preserved eGFR_predischarge_ (*n* = 51)	Impaired eGFR_predischarge_ (*n* = 68)	*p* Value
Age, years	71.2 ± 15.7	75.3 ± 10.1	0.086
Male, *n* (%)	39 (76.5%)	52 (76.5%)	>0.999
BMI, kg/m^2^	23.9 ± 4.1	24.0 ± 4.8	0.907
Smoking, *n* (%)	13 (25.5%)	14 (20.6%)	0.527
HTN, *n* (%)	38 (74.5%)	54 (79.4%)	0.527
DM, *n* (%)	13 (25.5%)	35 (51.5%)	0.004
AF, *n* (%)	27 (52.9%)	30 (44.1%)	0.340
Hyperlipidemia, *n* (%)	9 (17.6%)	20 (29.4%)	0.139
IHD, *n* (%)	20 (39.2%)	40 (58.8%)	0.034
HFrEF, *n* (%)	30 (58.8%)	29 (41.2%)	0.057
LVEF, %	38.8 ± 14.7	41.4 ± 16.1	0.543
Laboratory data			
HDL‐C, mg/dl	46.6 ± 21.1	40.3 ± 16.8	0.144
LDL‐C, mg/dl	84.4 ± 28.3	91.7 ± 31.8	0.261
hs‐CRP, mg/dl	1.1 ± 1.2	1.2 ± 1.6	0.711
Troponin‐I, ng/ml	0.1 ± 0.2	0.1 ± 0.2	0.940
NT‐pro‐BNP, pg/ml	3838.6 ± 4905.4	4115.7 ± 3403.2	0.782
Creatinine_admission_, mg/dl	1.2 ± 0.5	2.1 ± 0.9	<0.001
Creatinine_predischarge_, mg/dl	0.9 ± 0.2	1.9 ± 0.7	<0.001
eGFR_admission_, ml/min/1.73 m^2^	68.6 ± 27.0	37.2 ± 14.5	<0.001
eGFR_predischarge_, ml/min/1.73 m^2^	86.2 ± 28.5	39.0 ± 12.5	<0.001
eGFR change, ml/min/1.73 m^2^	0.4 ± 0.6	0.1 ± 0.4	0.012
Predischarge medication			
Diuretics, *n* (%)	36 (70.6%)	59 (86.8%)	0.030
ACEI/ARB, *n* (%)	39 (76.5%)	46 (67.6%)	0.292
Beta‐blockers, *n* (%)	25 (49.0%)	31 (45.6%)	0.711
Digitalis, *n* (%)	19 (37.3%)	8 (11.8%)	0.001
Vasodilators, *n* (%)	22 (43.1%)	41 (60.3%)	0.064
Follow‐up duration (years)	3.6 ± 3.7	1.9 ± 2.5	0.004

Abbreviations: ACEI, angiotensin‐converting enzyme inhibitor; AF, atrial fibrillation; ARB, angiotensin receptor blocker; BMI, body mass index; DM, diabetes mellitus; eGFR, estimated glomerular filtration rate; HDL‐C, high‐density lipoprotein‐cholesterol; HFrEF, heart failure with reduced ejection fraction; HTN, hypertension; hs‐CRP, high‐sensitivity C‐reactive protein; IHD, ischemic heart disease; LDL‐C, low‐density lipoprotein‐cholesterol; LVEF, left ventricular ejection fraction; NT‐pro‐BNP, N‐terminal pro‐brain natriuretic peptide.

Other biomarkers, cardiac troponin‐I, NT‐pro‐BNP, and hs‐CRP, were similar in patients with preserved or impaired renal function according to either eGFR_admission_ or eGFR_predischarge_.

### 3.2 Renal function changes during hospitalization

We further analyzed the changes in the eGFR between admission and discharge. There were 41 patients with eGFR decline and 78 without eGFR decline. When compared to those without eGFR decline, patients with eGFR decline used more diuretics (90.2% vs. 74.4%, *p* = .040) but less digitalis (12.2% vs. 28.2%, *p* = .048). The mean eGFR_admission_ was similar in the two groups. However, patients with eGFR decline had lower eGFR_predischarge_ than those without eGFR decline (46.8 ± 25.6 ml/min/1.73 m^2^ vs. 65.8 ± 32.2 ml/min/1.73 m^2^, *p* = .001). The changes in eGFR in the two groups were −0.2 ± 0.1 ml/min/1.73 and 0.4 ± 0.5 ml/min/1.73 m^2^ (*p* < .001), respectively (Supporting Information: Table 2). Cardiac troponin‐I, NT‐pro‐BNP, and hs‐CRP levels were similar in patients with and without eGFR decline.

### 3.3 Renal function and outcomes in patients with ADHF

During an average follow‐up period of 2.6 ± 3.2 years, 66 patients experienced 4P‐MACE, including 7 patients with CV death, 4 patients with nonfatal MI, 5 patients with nonfatal stroke, and 50 patients with nonfatal HF hospitalization. Patients with impaired eGFR_predischarge_ had more 4P‐MACE (*p* = .019) and HF hospitalization (*p* = .042) than those with preserved eGFR_predischarge_ (Table 3). Otherwise, the outcomes in patients with preserved eGFR_admission_ and impaired eGFR_admission_ were similar; the outcomes in patients with eGFR decline were also similar to those in patients without eGFR decline.

**Table 3:** Outcomes according to renal function

	Preserved eGFR_admission_	Impaired eGFR_admission_	*p* Value
CV death, *n* (%)	4 (11.4%)	3 (3.6%)	0.112
Nonfatal MI, *n* (%)	0 (0.0%)	4 (4.8%)	0.243
Nonfatal stroke, *n* (%)	0 (0.0%)	5 (6.0%)	0.169
Nonfatal HF, *n* (%)	12 (34.3%)	38 (45.2%)	0.270
4P‐MACE, *n* (%)	16 (45.7%)	50 (59.5%)	0.167

	**Preserved eGFR_predischarge_**	**Impaired eGFR_predischarge_**	***p* Value**
CV death, *n* (%)	4 (7.8%)	3 (4.4%)	0.343
Nonfatal MI, *n* (%)	1 (2.0%)	3 (4.4%)	0.424
Nonfatal stroke, *n* (%)	1 (2.0%)	4 (5.9%)	0.284
Nonfatal HF, *n* (%)	16 (31.4%)	34 (50.0%)	0.042
4P‐MACE, *n* (%)	22 (43.1%)	44 (64.7%)	0.019

	**No eGFR decline**	**eGFR decline**	***p* Value**
CV death, *n* (%)	5 (6.4%)	2 (4.9%)	0.544
Nonfatal MI, *n* (%)	2 (2.6%)	2 (4.9%)	0.428
Nonfatal stroke, *n* (%)	3 (3.8%)	2 (4.9%)	0.564
Nonfatal HF, *n* (%)	31 (39.7%)	19 (46.3%)	0.488
4P‐MACE, *n* (%)	41 (52.6%)	25 (61.0%)	0.248

Abbreviations: CV, cardiovascular; eGFR, estimated glomerular filtration rate; HF, heart failure; MACE, major adverse cardiovascular event; MI, myocardial infarction.

**Figure 1:**
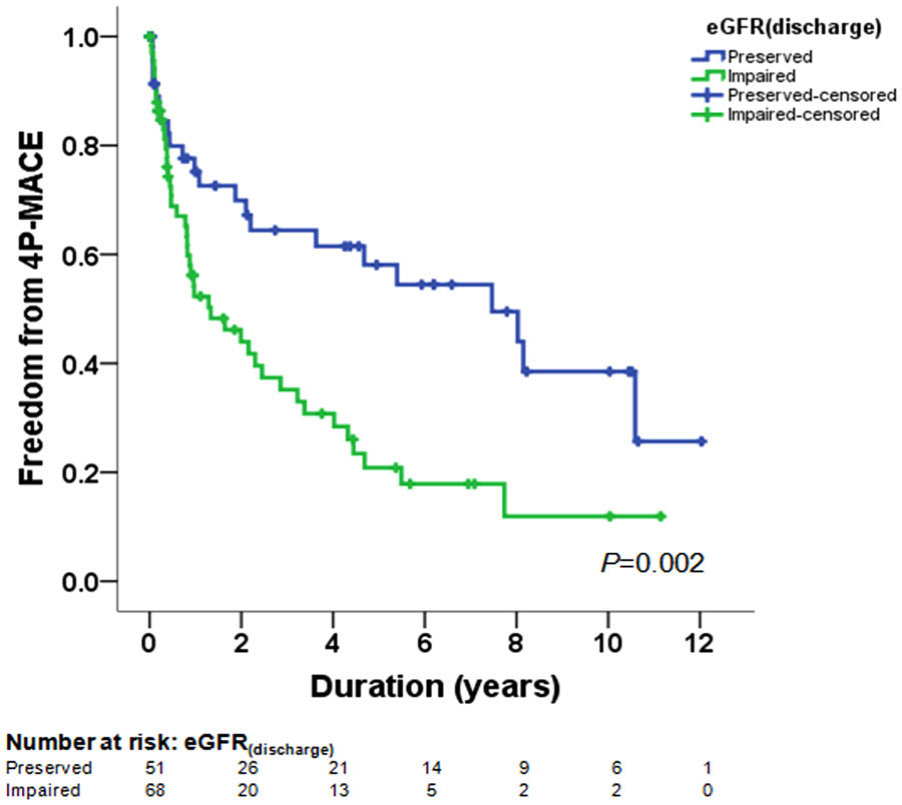
Kaplan–Meier survival curves showing the absence of 4P‐MACE according to eGFR_discharge_ in patients with ADHF. All participants were divided into two groups according to eGFR_predischarge_. The blue line represents the patient group with preserved eGFR_discharge_ (eGFR_predischarge_ ≥ 60 ml/min/1.73 m^2^). The green line represents the patient group with impaired eGFR_predischarge_ (eGFR_predischarge_ < 60 ml/min/1.73 m^2^). Differences were compared using the log‐rank test (*p* = .002). ADHF, acute decompensated heart failure; eGFR, estimated glomerular filtration rate; 4P‐MACE, 4P‐major adverse cardiovascular events.

The Kaplan–Meier survival curves and log‐rank test were used to identify the number of participants who did not develop 4P‐MACE during the follow‐up period. Although there was a trend of higher 4P‐MACE in patients with impaired eGFR_admission_ than in those with preserved eGFR_admission_, the difference was not significant (*p* = .125) (Supporting Information: Figure 1). The incidence of 4P‐MACE was significantly higher in patients with impaired eGFR_predischarge_ than in those with preserved eGFR_predischarge_ (*p* = .002; Figure 1). The incidence of 4P‐MACE was similar in patients with and without eGFR decline (*p* = .115) (Supporting Information: Figure 2).

Cox regression analysis revealed that in addition to age, impaired eGFR_predischarge_ was significantly correlated with the development of 4P‐MACE (HR, 2.003; 95% CI, 1.072–3.744; *p* = .029; Table 4). Neither impaired eGFR_admission_ nor eGFR decline between admission and discharge was associated with the development of 4P‐MACE.

**Table 4:** Multivariate Cox proportional hazard analysis

	HR	(95% CI)	*p* Value
Age, years	1.048	(1.023–1.074)	<0.001
Gender (male vs. female)	0.858	(0.461–1.596)	0.629
HTN (yes vs. no)	0.747	(0.394–1.415)	0.371
DM (yes vs. no)	1.390	(0.793–2.436)	0.250
IHD (yes vs. no)	1.626	(0.963–2.744)	0.069
ACEI/ARB (yes vs. no)	0.757	(0.407–1.408)	0.379
Beta‐blocker (yes vs. no)	1.504	(0.875–2.585)	0.139
Digitalis (yes vs. no)	1.179	(0.648–2.144)	0.590
Impaired GFR_admission_ (yes vs. no)	1.405	(0.751–2.627)	0.287
Age, years	1.046	(1.020–1.072)	<0.001
Gender (male vs. female)	0.870	(0.466–1.625)	0.662
HTN (yes vs. no)	0.879	(0.472–1.639)	0.686
DM (yes vs. no)	1.180	(0.664–2.098)	0.572
IHD (yes vs. no)	1.562	(0.933–2.613)	0.090
ACEI/ARB (yes vs. no)	0.751	(0.410–1.378)	0.355
Beta‐blocker (yes vs. no)	1.490	(0.866–2.563)	0.150
Digitalis (yes vs. no)	1.492	(0.786–2.832)	0.221
Impaired eGFR_predischarge_ (yes vs. no)	2.003	(1.072–3.744)	0.029
Age, years	1.047	(1.021–1.073)	<0.001
Gender (male vs. female)	0.875	(0.469–1.634)	0.676
HTN (yes vs. no)	0.862	(0.456–1.631)	0.649
DM (yes vs. no)	1.447	(0.832–2.517)	0.191
IHD (yes vs. no)	1.682	(1.007–2.808)	0.047
ACEI/ARB (yes vs. no)	0.740	(0.404–1.356)	0.330
Beta‐blocker (yes vs. no)	1.437	(0.838–2.464)	0.188
Digitalis (yes vs. no)	1.193	(0.650–2.188)	0.569
eGFR decline (yes vs. no)	1.257	(0.727–2.173)	0.414

Abbreviations: ACEI, angiotensin‐converting enzyme inhibitor; ARB, angiotensin receptor blocker; CI, confidence interval; DM, diabetes mellitus; eGFR, estimated glomerular filtration rate; HR, hazard ratio; HTN, hypertension; IHD, ischemic heart disease.

## 4 DISCUSSION

In this study, we found that impaired renal function before discharge was independently associated with poor clinical outcomes in Chinese patients with ADHF. However, WRF during hospitalization was not related to clinical outcomes in these patients.

### 4.1 Renal function impairment and outcomes in patients with ADHF

Renal function impairment, which is common in patients with ADHF, is associated with poor clinical outcomes in these patients.^4^, ^22^ In the CRIC study,^4^ focusing on a large US CKD population, the rate ratio for HF rehospitalization within 30 days was 2.6‐ and 1.9‐fold higher in eGFR 30–44 and <30 ml/min/1.73 m^2^, respectively, compared with eGFR ≥45 ml/min/1.73 m^2^. Heywood et al.^22^ reported that renal dysfunction at admission was associated with higher in‐hospital mortality in 118 465 patients hospitalized with ADHF. In the present study, we found that impaired renal function before discharge, defined as eGFR_predischarge_ < 60 ml/min/1.73 m^2^, was associated with poor outcomes in patients with ADHF, which were mostly driven by nonfatal HF. Although patients with impaired renal function had more comorbidities than those with preserved renal function, the findings were still consistent after adjusting for baseline comorbidities, including HTN, DM, and IHD. Interestingly, cardiac biomarkers, including troponin‐I and NT‐pro‐BNP, were similar in patients with preserved and impaired renal function. The findings suggest that renal function itself, rather than comorbidities or the severity of HF, is related to clinical outcomes in patients with ADHF.

### 4.2 Definitions of WRF

WRF is commonly observed in patients hospitalized for ADHF, either on admission or during hospitalization. The definitions of WRF vary among different studies. Some were defined by increased levels of creatinine^7^, ^9^, ^10^, ^11^, ^12^, ^14^, ^15^, ^16^; some were defined by an increased percentage of creatinine^8^; and some were defined by both.^5^, ^6^, ^13^ Recently, eGFR (calculated using the CKD‐EPI formula) has been used to define WRF.^17^, ^18^ In the present study, we used eGFR (calculated using the MDRD formula) to evaluate changes in renal function during hospitalization, which had better performance than creatinine and is commonly used in our clinical practice.^21^ Using this definition, WRF during hospitalization was noted in 41 (34.5%) of 119 patients, which meant that nearly two‐thirds of the patients could have preserved or improved renal function after decongestion therapy. Interestingly, WRF was not associated with comorbidities in these patients. However, it was related to more diuretic use and less digitalis use before discharge in these patients.

### 4.3 Impacts of WRF on clinical outcomes in patients with ADHF

The impact of WRF during hospitalization for ADHF has been examined in numerous studies. Some studies have reported that WRF is associated with worse long‐term outcomes. Gottlieb et al.^5^ reported that any WRF predicted increased in‐hospital mortality and prolonged hospital stays in patients hospitalized for HF. In the Vasodilation in the Management of Acute Congestive Heart Failure (VMAC) trial,^7^ WRF was associated with a higher rate of 6‐month mortality (37.9% vs. 18.8%, *p* < .001) and length of hospitalization (11.8 ± 9.1 vs. 8.3 ± 7.1 days, *p* < .001) in patients with HF. In the Organized Program to Initiate Lifesaving Treatment in Hospitalized Patients With Heart Failure (OPTIMIZE‐HF) registry,^9^, ^10^ WRF was associated with a higher rate of 30‐day readmission (21.8% vs. 20.6%; *p* = .01), 30‐day mortality (10.0% vs. 7.2%, *p* < .001), and 1‐year mortality (HR, 1.12; 95% CI, 1.04–1.20, *p* = .003) after HF admission. A prospective study of ADHF showed that patients with WRF had a poorer outcome, defined as rehospitalization and post‐discharge death, compared with patients without WRF (HR, 1.12; 95% CI, 1.02–1.22; *p* = .015).^11^ Berra et al.^12^ reported that WRF was strongly associated with a higher risk of death or readmission within 1 year after discharge in patients hospitalized for HF (HR, 1.24; 95% CI, 1.06–1.45; *p* = .0059). Other related studies have also suggested that HF patients with WRF were likely to have a prolonged length of hospital stay, increased healthcare costs, increased in‐hospital mortality, and higher rates of rehospitalization and post‐discharge death.^6^

In contrast, some studies have demonstrated that WRF is not necessarily associated with clinical outcomes in patients with HF. In a prospective multicenter study,^14^ patients with WRF had longer duration admissions, but a similar mortality and rehospitalization rate to those without WRF. In the Diuretic Optimization Strategies Evaluation (DOSE) trial,^15^ when under high‐dose diuretic treatment, WRF was not associated with the composite endpoint of death, rehospitalization, or emergency room visit within 60 days when compared with patients with stable renal function. In the PROTECT study (Placebo‐controlled Randomized Study of the Selective A1 Adenosine Receptor Antagonist Rolofylline for Patients Hospitalized with Acute Decompensated Heart Failure and Volume Overload to Assess Treatment Effect on Congestion and Renal Function study),^16^ WRF was found to be associated with longer length of admission and a higher risk of death or readmission for CV or renal reason within 30 days, only in patients who at the time of creatinine measurement were significantly congested. Using data from the Ultrafiltration in Decompensated Heart Failure with Cardiorenal Syndrome and DOSE trials,^17^ an in‐hospital decline in eGFR was not significantly associated with the composite outcome of death or rehospitalization within 60 days; however, a decline in eGFR may be associated with better outcomes when NT‐proBNP declined. Using data from the Efficacy of Vasopressin Antagonism in Heart Failure Outcome Study with Tolvaptan (EVEREST),^18^ acute declines in kidney function were associated with an increased risk of mortality and CV outcomes only in those patients who had worsened markers of decongestion. In this study, we found that WRF itself was not associated with CV death, nonfatal MI, nonfatal stroke, nonfatal HF hospitalization, or the composite endpoint of 4P‐MACE in patients admitted for ADHF. However, it remains unclear whether WRF results in impaired renal function before discharge, which is related to poor outcomes in these patients.

### 4.4 Study population and outcomes

Our study investigated the clinical outcome in patients hospitalized for ADHF. Since patients with ADHF were a highly heterogeneous population and were associated with multiple medical comorbidities,^23^, ^24^, ^25^ it was important to enroll appropriate study participants. To decrease the heterogeneity of the participants, we had excluded the patients with acute coronary syndrome, significant aortic valve disease, myocarditis, cardiomyopathy, and so on. Furthermore, to diminish the potential confounding factors for renal function and clinical outcomes, we also excluded patients who were comorbid with other chronic major organs disease. Finally, to investigate the influence of renal function changes on patients hospitalized for ADHF, patients with ESRD were also excluded. The renal functions of our participants upon admission (eGFR_admission_) and before discharge (eGFR_predischarge_) were 50.7 ± 25.9 ml/min/1.73 m^2^ and 59.2 ± 31.3 ml/min/1.73 m^2^, respectively.

Different from previous studies, which mainly focused on mortality and HF rehospitalization,^5^, ^6^, ^7^, ^8^, ^9^, ^10^, ^11^, ^12^, ^13^, ^14^, ^15^, ^16^ clinical outcome in this study was defined as 4P‐MACE. The composite endpoints of 3P‐MACE and/or 4P‐MACE are increasingly used in recent clinical trials, especially in those conducting in diabetic population.^26^, ^27^, ^28^ Since studies had shown that reduced eGFR was independently associated with increased risk of MACE regardless of diabetes status,^29^, ^30^ we adopted 4P‐MACE as our clinical outcome.

### 4.5 Race and ethnicity

Racial differences in HF outcomes have been reported in previous studies,^31^, ^32^, ^33^ which revealed that black and white patients had worse outcomes than other ethnicities. Data from the National Inpatient Sample (NIS) in the USA revealed that the age‐standardized HF hospitalization rate was highest in Blacks, followed by Hispanics, Whites, and Asian/Pacific Islanders; the inpatient mortality was highest for Whites.^31^ Data from the ARIC Community Surveillance Study^32^ showed that white patients had a significantly higher mortality at 1 year compared with black patients, and worse renal function served as an independent predictor of mortality in white patients. In a retrospective study involving 53 640 hospitalized HF patients,^33^ every increase in creatinine of 0.5 mg/dl was associated with a 10% increased risk in adjusted mortality for Blacks, compared with 15% increased risk in Whites.

The prevalence and incidence of CKD in Taiwan are relatively high compared to those in other countries, and it is associated with all‐cause mortality in Taiwan.^34^, ^35^ In this study, impaired renal function, either upon admission or before discharge, was commonly noted in our patients (84 [70.6%] and 68 [57.1%], respectively). We found that impaired renal function before discharge was associated with up to two times the risk of 4P‐MACE (HR, 2.003; 95% CI, 1.072–3.744; *p* = .029) in Chinese patients with ADHF. This finding is compatible with the risks reported in the CRIC study,^4^ which was mainly focused on non‐Hispanic Whites and non‐Hispanic Blacks. This suggests that the impact of renal dysfunction in Chinese patients is as important as that in white and black patients. Since the relationships between ADHF and renal dysfunction have rarely been reported in the Chinese population, our study provides important information about risk stratification in patients with ADHF.

### 4.6 Limitations

This study has some limitations. First, this was a single‐center study with a small study population. Since there were few studies to comprehensively investigate the relationship between renal function during hospitalization and clinical outcomes in Chinese patients with ADHF, our study provided valuable information. Further studies with larger sample sizes are required. Second, we did not have information regarding baseline renal function before admission. Since most of the patients were newly diagnosed with HF and were experiencing their first hospitalization, they did not undergo any tests before admission. Furthermore, the study was designed to investigate renal function during hospitalization and clinical outcomes in patients with ADHF; only renal function tests during hospitalization were collected. The findings are more applicable to patients without previous hospital visits, experiencing their first HF hospitalization. Third, serum biomarkers, troponin‐I, NT‐pro‐BNP, and hs‐CRP, were only measured once in this study. Therefore, we did not have information regarding the change in NT‐pro‐BNP, which was used as a marker of decongestion in previous studies.^18^ Further studies are needed to clarify the impact of biomarkers and their changes on clinical outcomes in this population.

## 5 CONCLUSIONS

Impaired renal function before discharge is a significant risk factor for poor clinical outcomes in Chinese patients with ADHF. However, WRF was not associated with clinical outcomes in these patients. Our study not only highlights the importance of renal function before discharge as a biomarker for risk stratification but also supports the safety of decongestion therapy for renal function in Chinese patients with ADHF.

## AUTHOR CONTRIBUTIONS

Hao‐Wei Lee contributed to conception and design, analysis and interpretation of data, and drafted the manuscript. Chih‐Yu Yang, Hsin‐Bang Leu, Po‐Hsun Huang, Tao‐Cheng Wu, Shing‐Jong Lin, and Jaw‐Wen Chen contributed to data acquisition and drafted the manuscript. Chin‐Chou Huang contributed to conception, data acquisition, analysis and interpretation of data, drafted and critically revised the manuscript. All authors gave final approval and agreed to be accountable for all aspects of work ensuring integrity and accuracy.

## CONFLICT OF INTEREST

The authors declare no conflict of interest.

## Supporting information

Supporting Information.

Supporting Information.

Supplementary information.

## Data Availability

The datasets generated during and/or analyzed during the current study are available from the corresponding author on reasonable request.
