# Supplementary figures and images for: Renal function during hospitalization and outcome in Chinese patients with acute decompensated heart failure: A retrospective study and literature review
Source: Clin Cardiol. 2022 Nov 7;46(1):57–66. doi: 10.1002/clc.23934 (PMC9849437; doi:10.1002/clc.23934)

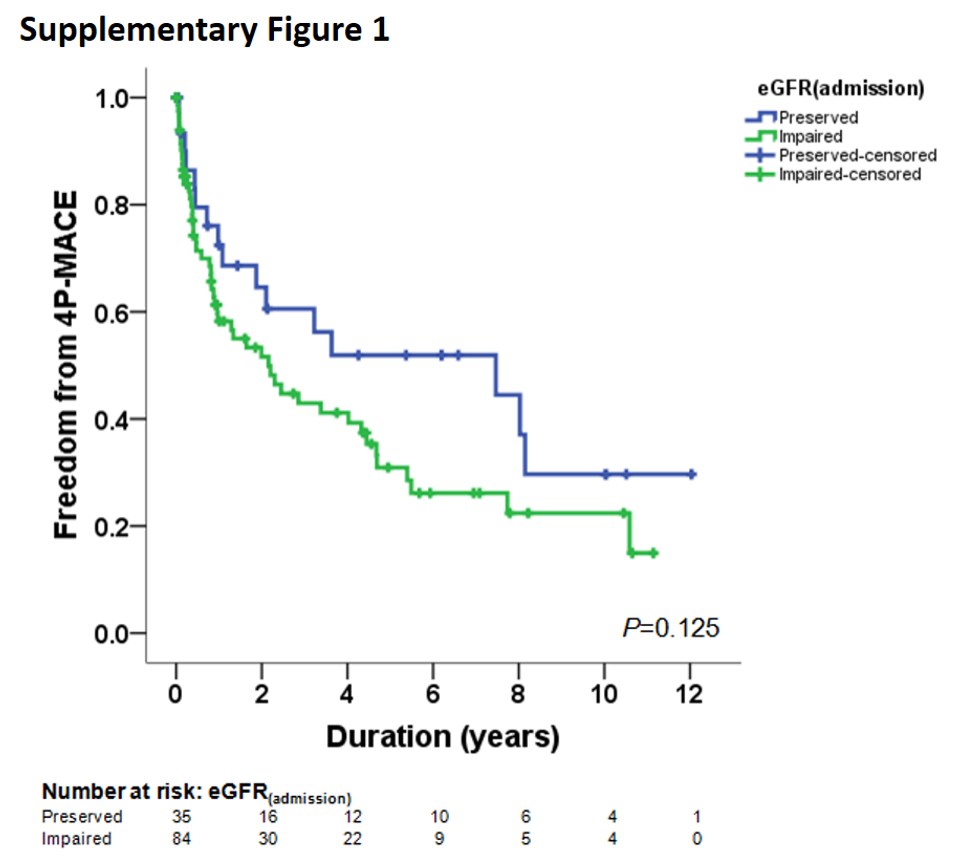

Supplement: Supplementary file 1 — Supporting Information. [file CLC-46-57-s002.tif]

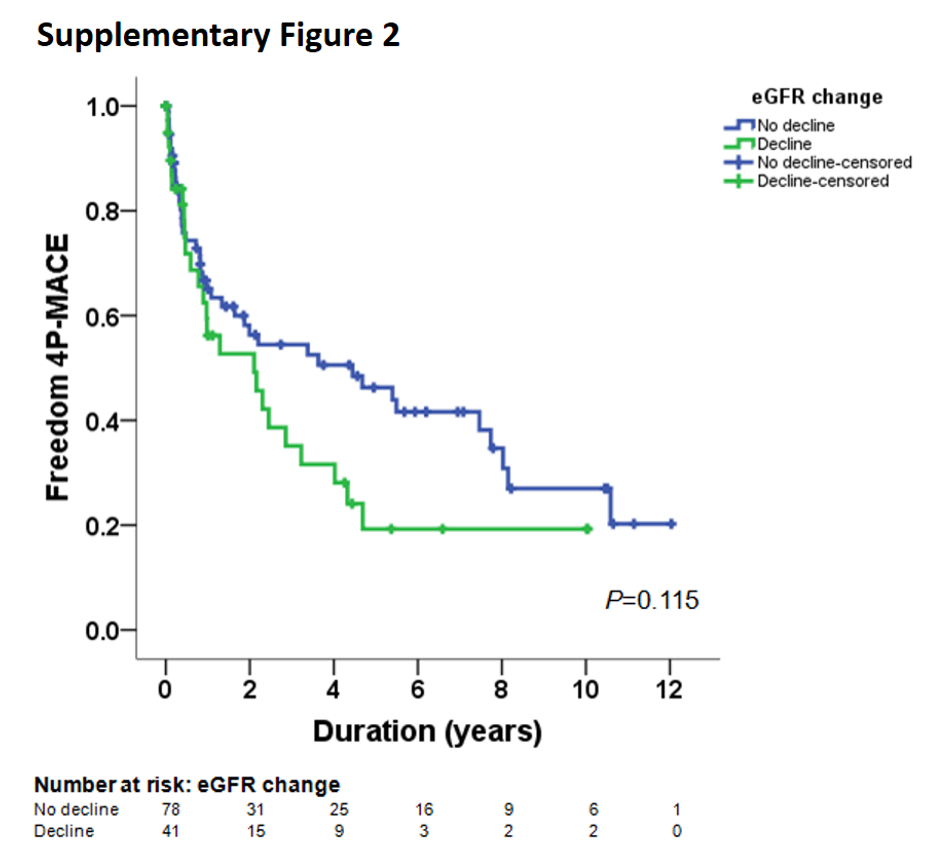

Supplement: Supplementary file 2 — Supporting Information. [file CLC-46-57-s003.tif]
