# Supplementary material for: Renal function during hospitalization and outcome in Chinese patients with acute decompensated heart failure: A retrospective study and literature review
Source: Clin Cardiol. 2022 Nov 7;46(1):57–66. doi: 10.1002/clc.23934 (PMC9849437; doi:10.1002/clc.23934)
Supplement: Supplementary file 3 — Supplementary information. [file CLC-46-57-s001.docx]

**Supplementary table 1 Baseline characteristics according to admission renal function**

|  | **Preserved eGFR_admission_**  **(n=35)** | **Impaired eGFR_admission_**  **(n=84)** | ***P*-value** |
| --- | --- | --- | --- |
| Age, years | 71.8 ± 13.7 | 74.3 ± 12.5 | 0.357 |
| Male, n(%) | 26 (74.3%) | 65 (77.4%) | 0.717 |
| BMI, kg/m^2^ | 22.6 ± 2.7 | 24.4 ± 4.9 | 0.131 |
| Smoking, n(%) | 8 (22.9%) | 19 (22.6%) | 0.977 |
| HTN, n(%) | 22 (62.9%) | 70 (83.3%) | 0.015 |
| DM, n(%) | 8 (22.9%) | 40 (47.6%) | 0.012 |
| AF, n(%) | 20 (57.1%) | 37 (44.0%) | 0.193 |
| Hyperlipidemia, n(%) | 5 (14.3%) | 24 (28.6%) | 0.098 |
| IHD, n(%) | 12 (34.3%) | 48 (57.1%) | 0.023 |
| HFrEF, n(%) | 21 (60.0%) | 37 (44.0%) | 0.113 |
| LVEF, % | 36.5 ± 15.7 | 41.7 ± 15.3 | 0.111 |
| **Laboratory data** |  |  |  |
| HDL-C, mg/dL | 47.4 ± 21.0 | 41.3 ± 18.0 | 0.212 |
| LDL-C, mg/dL | 84.4 ± 28.5 | 90.2 ± 31.1 | 0.398 |
| hs-CRP, mg/dL | 1.1 ± 1.2 | 1.2 ± 1.5 | 0.732 |
| Troponin-I, ng/mL | 0.2 ± 0.2 | 0.1 ± 0.2 | 0.385 |
| NT-pro-BNP, pg/ml | 4112.1 ± 5965.6 | 3971.5 ± 3232.3 | 0.921 |
| Creatinine_admission_, mg/dL | 1.0 ± 0.2 | 2.0 ± 0.8 | <0.001 |
| Creatinine_predischarge_, mg/dL | 0.9 ± 0.3 | 1.7 ± 0.7 | <0.001 |
| eGFR_admission_, mL/min/1.73m^2^ | 81.3 ± 23.1 | 37.9 ± 13.3 | <0.001 |
| eGFR_predischarge_, mL/min/1.73m^2^ | 89.5 ± 32.5 | 46.6 ± 20.4 | <0.001 |
| eGFR change, mL/min/1.73m^2^ | 0.1 ± 0.3 | 0.3 ± 0.6 | 0.065 |
| **Predischarge medication** |  |  |  |
| Diuretics, n(%) | 29 (82.9%) | 66 (78.6%) | 0.595 |
| ACEI/ARB, n(%) | 29 (82.9%) | 56 (66.7%) | 0.075 |
| Beta-blockers, n(%) | 17 (48.6%) | 39 (46.4%) | 0.831 |
| Digitalis, n(%) | 12 (34.3%) | 15 (17.9%) | 0.051 |
| Vasodilators, n(%) | 13 (37.1%) | 50 (59.5%) | 0.026 |
| **Follow-up duration (years)** | 3.3 ± 3.7 | 2.3 ± 2.9 | 0.126 |

ACEI, angiotensin-converting enzyme inhibitor; AF, atrial fibrillation; ARB, angiotensin receptor blocker; BMI, body mass index; DM, diabetes mellitus; eGFR, estimated glomerular filtration rate; HDL-C, high-density lipoprotein-cholesterol; HFrEF, heart failure with reduced ejection fraction; HTN, hypertension; hs-CRP, high sensitivity C-reactive protein; IHD, ischemic heart disease; LDL-C, low-density lipoprotein-cholesterol; LVEF, left ventricular ejection fraction; NT-pro-BNP, N-terminal pro-brain natriuretic peptide.

**Supplementary table 2 Baseline characteristics according to renal function changes during hospitalization**

|  | **No eGFR decline (n=78)** | **eGFR decline (n=41)** | ***P*-value** |
| --- | --- | --- | --- |
| Age, years | 72.4 ± 13.8 | 75.8 ± 10.8 | 0.144 |
| Male, n(%) | 60 (76.9%) | 31 (75.6%) | 0.872 |
| BMI, kg/m^2^ | 24.3 ± 4.9 | 23.2 ± 3.6 | 0.239 |
| Smoking, n(%) | 16 (20.5%) | 11 (26.8%) | 0.434 |
| HTN, n(%) | 61 (78.2%) | 31 (75.6%) | 0.748 |
| DM, n(%) | 33 (42.3%) | 15 (36.6%) | 0.545 |
| AF, n(%) | 39 (50.0%) | 18 (43.9%) | 0.527 |
| Hyperlipidemia, n(%) | 17 (21.8%) | 12 (29.3%) | 0.367 |
| IHD, n(%) | 36 (46.2%) | 24 (58.5%) | 0.199 |
| HFrEF, n(%) | 40 (51.3%) | 18 (43.9%) | 0.444 |
| LVEF, % | 40.7 ± 14.9 | 39.5 ± 16.8 | 0.720 |
| **Laboratory data** |  |  |  |
| HDL-C, mg/dL | 40.5 ± 18.3 | 49.1 ± 19.6 | 0.065 |
| LDL-C, mg/dL | 84.3 ± 28.8 | 96.5 ± 32.0 | 0.089 |
| hs-CRP, mg/dL | 1.1 ± 1.1 | 1.4 ± 2.0 | 0.256 |
| Troponin-I, ng/mL | 0.1 ± 0.1 | 0.2 ± 0.3 | 0.059 |
| NT-pro-BNP, pg/ml | 3554.± 3003.9 | 4861.5 ± 5453.0 | 0.247 |
| Creatinine_admission_, mg/dL | 1.8 ± 0.9 | 1.5 ± 0.7 | 0.092 |
| Creatinine_predischarge_, mg/dL | 1.3 ± 0.6 | 1.8 ± 0.8 | 0.002 |
| eGFR_admission_, mL/min/1.73m^2^ | 48.2 ± 24.8 | 55.4 ± 27.6 | 0.170 |
| eGFR_predischarge_, mL/min/1.73m^2^ | 65.8 ± 32.2 | 46.8 ± 25.6 | 0.001 |
| eGFR change, mL/min/1.73m^2^ | 0.4 ± 0.5 | -0.2 ± 0.1 | <0.001 |
| **Predischarge medication** |  |  |  |
| Diuretics, n(%) | 58 (74.4%) | 37 (90.2%) | 0.040 |
| ACEI/ARB, n(%) | 54 (69.2%) | 31 (75.6%) | 0.464 |
| Beta-blockers, n(%) | 36 (46.2%) | 20 (48.8%) | 0.785 |
| Digitalis, n(%) | 22 (28.2%) | 5 (12.2%) | 0.048 |
| Vasodilators, n(%) | 39 (50.0%) | 24 (58.5%) | 0.375 |
| **Follow-up duration (years)** | 2.9 ± 3.4 | 2.0 ± 2.5 | 0.106 |

ACEI, angiotensin-converting enzyme inhibitor; AF, atrial fibrillation; ARB, angiotensin receptor blocker; BMI, body mass index; DM, diabetes mellitus; eGFR, estimated glomerular filtration rate; HDL-C, high-density lipoprotein-cholesterol; HFrEF, heart failure with reduced ejection fraction; HTN, hypertension; hs-CRP, high sensitivity C-reactive protein; IHD, ischemic heart disease; LDL-C, low-density lipoprotein-cholesterol; LVEF, left ventricular ejection fraction; NT-pro-BNP, N-terminal pro-brain natriuretic peptide.
